# Recurrence of an Immature Thyroid Infiltrating Teratoma in a Female Infant

**DOI:** 10.1055/a-2733-2840

**Published:** 2025-11-11

**Authors:** Nabila Bouzakri, Ann-Kathrin Lederer, Julia I. Staubitz-Vernazza, Larissa Seidmann, Joachim Pohlenz, Meinolf Siepermann, Thomas J. Musholt

**Affiliations:** 1 Department of General, Visceral and Transplant Surgery of the University Medical Center of the Johannes Gutenberg University Mainz, Mainz, Germany; 2 Institute of Pathology of the University Medical Center of the Johannes Gutenberg University Mainz, Mainz, Germany; 3 Department of Clinic and Polyclinic for Pediatrics and Adolescent Medicine, Johannes Gutenberg Universitat Mainz, Mainz, Rheinland-Pfalz, Germany; 4 Clinic for Pediatrics and Adolescent Medicine, Kinderkrankenhaus Amsterdamer Strasse, Cologne, NRW, Germany

**Keywords:** teratoma, rare diseases, thyroid gland, dermoid cyst, newborn

## Abstract

This case report describes the recurrence of an immature thyroid infiltrating teratoma in a female infant. Initially treated surgically for a cervical mass, the teratoma recurred, requiring further intervention. The case highlights the importance of multidisciplinary care and long-term follow-up in managing complex pediatric neoplasms.

## Introduction

Teratomas are an inhomogeneous group of germ cell tumors located at different body sites that can occur at any age and in both sexes. Teratomas are divided into gonadal teratomas (testes and ovaries) and extragonadal teratomas, which are classified according to their localization. 1 Typical localizations are cervical, intracranial, craniofacial, mediastinal, gastric, retroperitoneal, and sacrococcygeal, with sacrococcygeal being the most common extra-gonadal localization. 1 2 3 4 Extragonadal teratomas appear to be more frequent in infants and children, whereas gonadal teratomas are a typical disease of sexually mature women. 5 The most common type of teratoma is the mature cystic teratoma of the ovary, also known as dermoid cyst. 6 Even though more than 95% of teratomas show a benign growth behavior, they can be life-threatening due to their localization. 4 7 8 The particular location of the cervical teratoma can cause obstruction of the esophagus and trachea, which is incompatible with life, especially in infants. 9 The compression can lead to an inability to breathe after birth resulting in rapid death of a newborn. 7 8 10 Depending on the amount of respiratory insufficiency, affected children usually undergo emergency or prompt surgery shortly after birth, in which the tumor is completely removed. 11 Due to the benign nature of the tumor, excellent results are reported after complete surgical removal without subsequent recurrence. 7 12 However, with this report we would like to discuss a case of a female infant in which, despite the macroscopically previous complete resection of congenital cervical teratoma, a rapid recurrence occurred that caused shortness of breath and threatened the patient's life.

## Case Presentation

Based on the recommendation of the CARE guidelines, 13 this report presents the clinical course of a female infant with a recurrent symptomatic cervical teratoma who underwent surgery at the Department of General, Visceral and Transplantation Surgery of the University Medical Center of Mainz, Germany.

### Chief Complaints

A 2-month-old eutrophic female infant (5.7 kg—89th percentile, 62 cm—98th percentile) suffering from progressive inspiratory stridor was transferred from another hospital to the Department of General, Visceral and Transplantation Surgery of the University Medical Center of Mainz for resection of recurrent cervical teratoma.

### History of Present Illness

The patient presented to an external pediatric department immediately after birth with a left-sided cervical mass and massive stridor. This was followed by immediate fiberoptic intubation and cervical surgery on the 6th day of life. Prior to surgery, the thyroid gland could not be differentiated from the tumor by imaging. Initial α-fetoprotein (AFP) was 41,126 ng/mL (normal range for newborns up to 100,000 ng/mL). During the operation, the encapsulated tumor was removed without any problems and the thyroid gland was preserved. Histological examination revealed the diagnosis of a mixed teratoma with mature and more than 50% immature parts, which was classified as grade 3 according to the classification of Frank Gonzalez-Crussi. 14 Parts of the teratoma extended to the resection margin. External interdisciplinary tumor board recommended a wait-and-see approach. The patient presented in an excellent condition 4 weeks after surgery. The progression of AFP was age-appropriate. The left lobe of the thyroid gland showed a single, echogenic nodule (10 × 7 × 9 mm) on imaging. After 4 weeks, the patient developed severe stridor due to a progressive cervical tumor, which led to her transfer to our hospital. AFP was still decreasing (387.8 ng/mL; normal range for infants younger than 4 months is up to 1,000 ng/mL).

### History of Past Illness and Personal and Family History

Parents denied chronic pre-existing conditions and allergies. There was no need for a long-term medication.

### Physical Examination

Clinical condition was slightly impaired due to dyspnea and eutrophic nutritional status. Color was pale pinkish. Breathing was spontaneous, no respiratory assistance. There was a prominent mass in the area of the anterior neck.

Cardiac: Restlessness with tachycardia up to 190/min, regular rhythm without murmurs, gallops or rubs.
Pulmonary: Inspiratory stridor, tachydyspnea with sternal and intercostal retractions, especially during restlessness, clear to auscultation bilaterally.
Abdomen: No abdominal pain, no tenderness, no resistance, regular peristalsis.
Neurology: Age-appropriate, social smile, grossly orienting unremarkable.

### Relevant Laboratory Values

Tumor marker AFP : 387 ng/mL.

The other laboratory values were within the normal range for the age group.

### Imaging Examinations

**Fig. 1:**
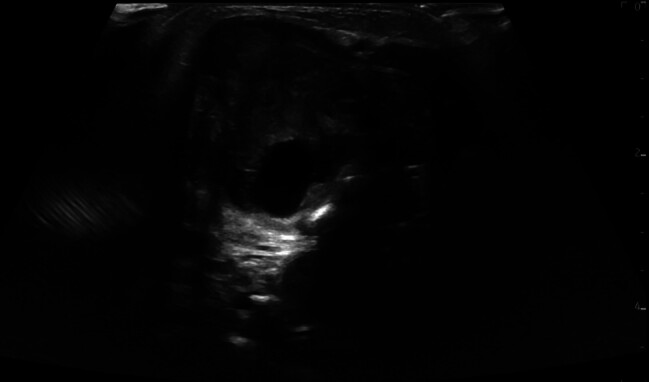
Ultrasound of the left anterior neck revealed an inhomogeneous mass.

Ultrasound results:Fig. 1 shows a characteristic picture of the findings of the ultrasound of the left anterior neck. The tumor volume was estimated at 16 mL by sonography.
MRI results: Clear progression of the originally resected tumor to the edge of the thymus; the left thyroid lobe cannot be delimited; compression of the trachea, displacing to a diameter of 2.3 mm in the upper part.

### Treatment

**Fig. 2:**
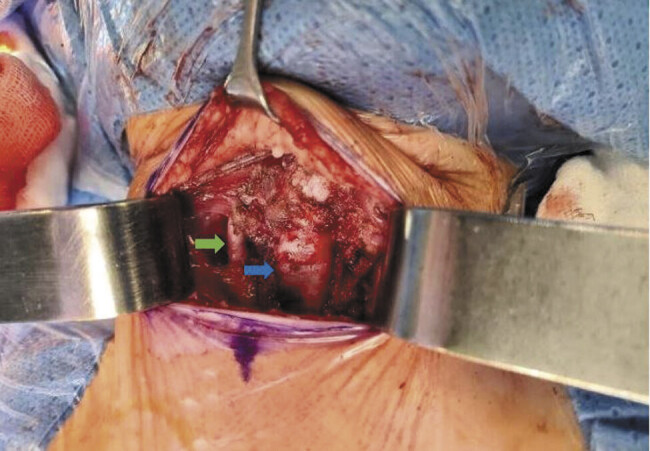
Intraoperative situs of the patient after resection of the recurrent tumor mass. Green arrow shows A. carotis communis right. Blue arrow shows trachea with anterolateral compression due to the tumor mass and tracheomalacia.

The patient's case was previously discussed in our interdisciplinary tumor board. The recommendation was a surgical resection as soon as possible due to the progressive respiratory distress. We performed an en-bloc tumor resection with thyroid isthmus and parts of the right and left thyroid lobe, shaving of the trachea, and a resection of the infrahyoid muscles ( Fig. 2 ). In accordance with the standards of our department we performed continuous intraoperative neuromonitoring (CIONM) using surface electrode-based endotracheal tube. For this purpose, we had to cut the attachable surface electrodes to fit a size 3.5 tube. We were able to derive regular neuromonitoring signals during the entire procedure. No damage to the vocal cord nerves was recorded at the end of the operation.

### Pathological Examination

**Fig. 3:**
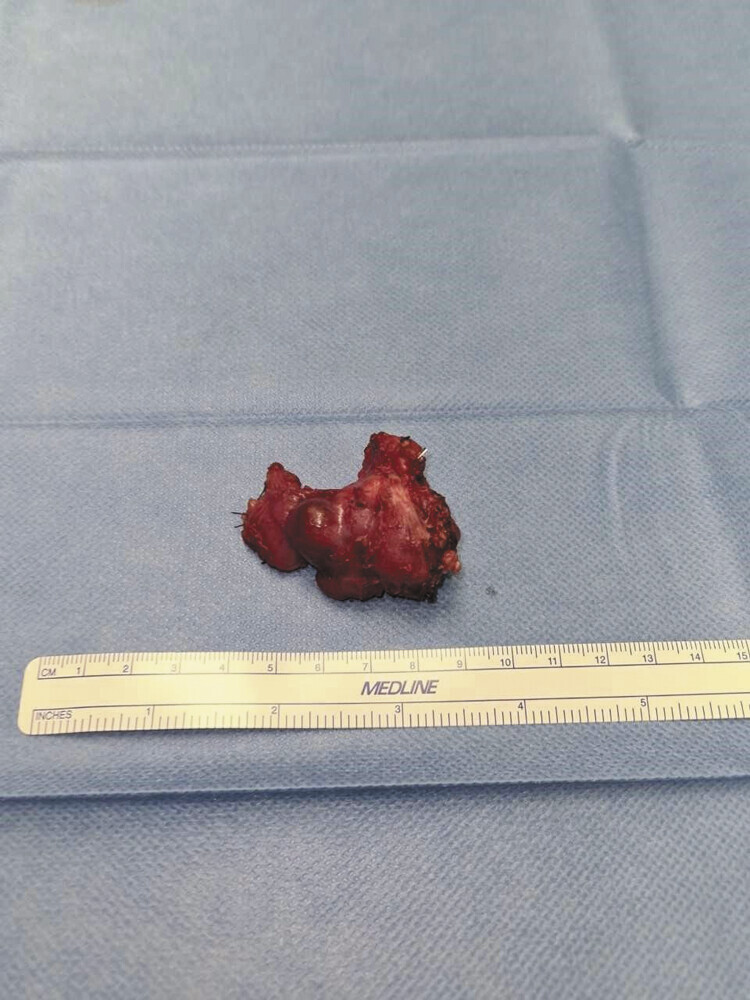
Macroscopic appearance of the mass after complete resection. The mass measured 5.0 × 3.5 × 3.0 cm and weighed 14 g.

**Fig. 4:**
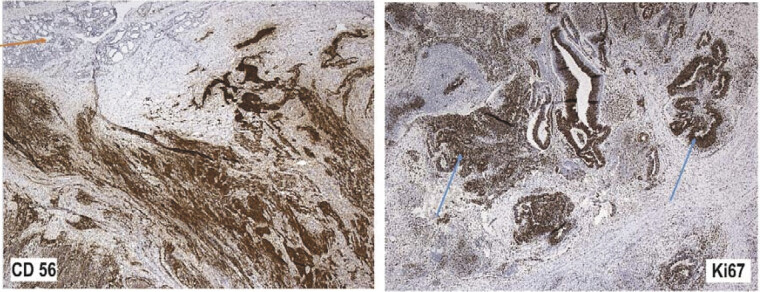
Histological findings (hematoxylin and eosin stain, HE) with typical thyroid structures (orange arrow) and infiltration of the immature teratoma (blue arrows).

After lamination, a maximum 2.8-cm large focal lesion with a soft, beige-brown cut surface is visible. The focal lesion partially extends up to 0.2 cm to the resection margin marked with ink ( Fig. 3 ). Histologically, fibrosed soft tissue as well as parts of the thyroid gland without a recognizable capsule can be recognized (see Fig. 4 ). Sparsely included thymus tissue with small focal epithelioid structures can be found. In addition to epithelial structures (approximately 5% of the tumor), mesenchymal structures such as immature cartilage tissue and connective tissue (approximately 15% of the tumor) can be seen. Furthermore, predominantly maturing glial tissue and plexus parts can be recognized. In places, tumor tissue extends directly into the inked preparation surface. There are three tumor-free lymph nodes.

**Fig. 5:**
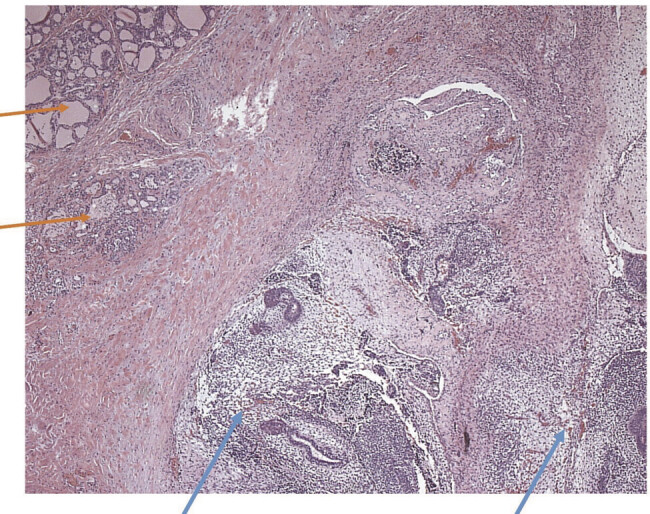
Findings of immunohistochemistry with typical thyroid structures (orange arrows) and infiltration of the immature teratoma (blue arrows).

Immunohistochemically, the tumor cells show a focal positivity for AFP and glypican-3, CD56, S-100, GEFAP, synaptophysin, SAL4, myogenin, SSTRA2, chromogranin, NSE, and total cytokeratin. We found a negativity for β-hCG and a small focal positivity for ALK1A4. Ki67 was significantly increased in epithelial and partially mesenchymal tumor parts (see Fig. 5 ).

### Outcome and Follow-up

There were no postoperative complications. AFP value measured postoperatively was 110 ng/mL. Due to pre-existing tracheomalacia with stridor, the patient was cared for in our pediatric intensive care unit for a total of 21 days. Continuous positive airway pressure (CPAP) therapy with Heliox (a mixture of helium and oxygen to improve the airflow and to relieve breathing distress) was initially administered. This quickly showed an improvement over the course of the hospitalization, and weaning of CPAP therapy was possible. With intermittent CPAP therapy without additional oxygen requirement, the patient was finally transferred to a hospital near her home. After a further 1-month stay in hospital, the patient was discharged home. In the meantime, the child is doing very well. She is developing in line with her age and is already walking. At a sonographic check-up 1 year after the operation, there is no indication of a local recurrence. Follow-up care is still continued.

## Discussion and Conclusion

To date, only a few cases of cervical teratoma have been published. Our case is the first in which a rapid recurrence developed after a previous complete removal.

The term “teratoma” was introduced by Rudolf Virchow more than 150 years ago and is derived from the Greek word τερας, which means monster. 15 Teratomas exist in an immature and a mature form. The mature form is capable of forming hair, teeth, and skin, among other things, which gave the teratoma its characteristic name. Mature teratomas are typically benign. Histologically, teratomas are composed of all three germ cell layers (endoderm, mesoderm, and ectoderm). 7 As mentioned in the introduction, teratomas can occur at any age and in different parts of the body. The exact incidence of infantile cervical teratomas is unclear, but it is estimated that cervical teratomas occur in approximately 1 in 20,000 to 40,000 live births. 2 16 About 6% of all teratomas are located cervically, which makes them a rare disease. 5 9 There are much more common differential diagnoses for cervical tumors such as thyroglossal duct or branchial cleft cysts as well as lymphatic malformations, but prenatal diagnosis can be essential to save a child's life. 7 9 In case of large tumors that obstruct the airways, the so-called EXIT (Ex Utero Intrapartum Treatment) approach uses the placental support after birth to ensure the oxygen supply until airways are stabilized. 17 18 It has been known for decades that surgical resection is the treatment of choice in case of cervical teratoma. In 1978, Rosenfeld et al published a case report in which they described how a 33-hour-old newborn had to undergo transverse neck incision due to increasing stridor caused by a cervical tumor, which was later diagnosed as teratoma with mature and immature elements. 12 At the time, colleagues emphasized that surgical removal is associated with an excellent outcome of infants affected. Bergé et al agreed with this assumption years later. 7 Bergé et al reported a case of a 12 cm × 9 cm × 7 cm congenital teratoma that was surgically removed on the second day of life. The child survived, and there was no recurrence of the disease. 7 Teratomas are usually benign tumors, and less than 5% of teratomas show a malignant growth behavior, which mostly affects adults. 7 8 19 Nevertheless, the complete resection of the teratoma appears to be of crucial importance for patient's survival. In 1985, Ernest Leck described the outcome of patients with various infantile craniocervical teratomas. 4 Interestingly, all patients with cervical teratomas survived, whereas most of the patients with an oropharyngeal or nasopharyngeal localization died due to irresectability of the tumor. In 2004, Hasiotou et al presented five cases of cervicocranial teratoma that supported the observation of Ernest Leck. 19 Malhotra et al assume that less than 10% of cervical teratomas recur depending on the resection and maturity status. 20 In this respect, our case illustrates that a macroscopically complete resection that histologically reaches the resection margins may not be sufficient to prevent recurrence despite the actually benign growth behavior of teratomas. Dharmarajan et al focused on mature und immature pediatric head and neck teratomas in their 15-year-period review that included 9 patients with immature teratomas and 11 patients with mature teratomas. 9 They distinguish differences between mature and immature pediatric head and neck teratomas in terms of their presentations, outcomes, management strategies, and potential for recurrence. Furthermore, Dharmarajan et al describe higher AFP serum levels in immature teratomas. The role of AFP levels in pediatric head and neck teratomas is not definitely established in the literature. AFP is often elevated in malignant teratomas, particularly those that contain yolk sac tumor elements.

These tumors, also known as endodermal sinus tumors, are aggressive and produce AFP.

As described in our report, the AFP level was more than 30 times higher before resection and fell significantly afterwards. Therefore, the AFP levels can be used to monitor the effectiveness of treatment in patients with malignant teratomas, most likely even if they are in the normal range. Nonetheless, the surveillance of AFP levels for predicting recurrence compared with traditional imaging and clinical monitoring needs to be explored in future prospective studies.

Overall, immature teratomas seem to occur more frequently in the anterior neck region and often require EXIT procedure. To our knowledge, the teratoma of our patient was not known before birth, and led to an emergency fiberoptic intubation after birth and to a transfer to an external pediatric intensive care unit. The prenatal diagnosis of a cervical teratoma can be difficult, but, fortunately, it was still possible to save the patient's life. The further course of the patient with a complete resection of the tumor is the recommended procedure in cervical teratoma and the only way to cure the patient. In previous publications, recurrence was noted in only one case of immature teratoma after incomplete resection (follow-up 51 months). 9 When the patient was transferred to our clinic for further surgical treatment, she presented with a pronounced inspiratory stridor with tachyspnoea and compression of the trachea by the recurrent tumor mass. Thus, the surgical intervention had to be performed promptly. The operation became more difficult because it was a revision, and revisional surgery is often more challenging than primary interventions. To achieve an en bloc resection of the recurrent tumor mass with superficial invasion of the trachea, we performed a tracheal shave excision. Postoperatively, the patient continued to struggle with breathing due to the previous compression of the trachea. Nevertheless, it was possible to accompany the patient on the road to recovery with a targeted intensive medical therapy. Our case illustrates the importance of interdisciplinary cooperation (also between hospitals, as we were in close contact with the transferring external hospital). The results of Dharmarajan et al and our case also underline how important it is for such patients to have surgery in an experienced center. 9 Apart from a scar, our patient has no vocal cord palsy due to a recurrent laryngeal nerve damage and no permanent damage of the trachea or the esophagus. So far, the follow-up examinations have been unremarkable, so we hope that the patient will continue to develop well and can grow up without any worries. As a consequence for the future, we recommend that unclear cervical masses in children, even in infants or newborns, should be presented at an experienced center for complex cervical surgery. If a cervical mass is suspected prenatally, we recommend that the delivery should also take place at a suitable center.

## Notes

Importance for the Pediatric Surgeon This case is particularly rare because the immature thyroid teratoma recurred rapidly despite seemingly complete resection, highlighting the tumor's unpredictable nature. It underscores the importance of vigilant long-term follow-up and multidisciplinary management in pediatric tumors.

## References

[1] Paradis J Koltai P J Pediatric teratoma and dermoid cysts Otolaryngol Clin North Am 2015 48 01 121 136 25439551 10.1016/j.otc.2014.09.009

[2] Harms D Zahn S Göbel U Schneider D T Pathology and molecular biology of teratomas in childhood and adolescence Klin Padiatr 2006 218 06 296 302 17080330 10.1055/s-2006-942271

[3] Grosfeld J L Billmire D F Teratomas in infancy and childhood Curr Probl Cancer 1985 9 09 1 53 10.1016/s0147-0272(85)80031-3 2415302

[4] Lack E E Extragonadal germ cell tumors of the head and neck region: review of 16 cases Hum Pathol 1985 16 01 56 64 2982716 10.1016/s0046-8177(85)80214-8

[5] Barksdale E M Jr Obokhare I Teratomas in infants and children Curr Opin Pediatr 2009 21 03 344 349 19417664 10.1097/MOP.0b013e32832b41ee

[6] Saba L Guerriero S Sulcis R Virgilio B Melis G Mallarini G Mature and immature ovarian teratomas: CT, US and MR imaging characteristics Eur J Radiol 2009 72 03 454 463 18804932 10.1016/j.ejrad.2008.07.044

[7] Bergé S J von Lindern J J Appel T Braumann B Niederhagen B Diagnosis and management of cervical teratomas Br J Oral Maxillofac Surg 2004 42 01 41 45 14706299 10.1016/s0266-4356(03)00174-8

[8] Shine N P Sader C Gollow I Lannigan F J Congenital cervical teratomas: diagnostic, management and postoperative variability Auris Nasus Larynx 2006 33 01 107 111 16168588 10.1016/j.anl.2005.07.003

[9] Dharmarajan H Rouillard-Bazinet N Chandy B M Mature and immature pediatric head and neck teratomas: a 15-year review at a large tertiary center Int J Pediatr Otorhinolaryngol 2018 105 43 47 29447817 10.1016/j.ijporl.2017.11.031

[10] Jordan R B Gauderer M WL Cervical teratomas: an analysis. Literature review and proposed classification J Pediatr Surg 1988 23 06 583 591 3047360 10.1016/s0022-3468(88)80373-7

[11] Olivares E Castellow J Khan J Grasso S Fong V Massive fetal cervical teratoma managed with the ex utero intrapartum treatment (EXIT) procedure Radiol Case Rep 2018 13 02 389 391 29904479 10.1016/j.radcr.2017.12.011 PMC5999839

[12] Rosenfeld C R Coln C D Duenhoelter J H Fetal cervical teratoma as a cause of polyhydramnios Pediatrics 1979 64 02 176 179 382080

[13] Riley D S Barber M S Kienle G S CARE guidelines for case reports: explanation and elaboration document J Clin Epidemiol 2017 89 218 235 28529185 10.1016/j.jclinepi.2017.04.026

[14] Gonzalez-Crussi F Extragonadal teratomas. Atlas of Tumor Pathology. 2nd series, fascicle 18 Washington, DC Armed Forces Institute of Pathology 1982

[15] Pantoja E Noy M A Axtmayer R W Colon F E Pelegrina I Ovarian dermoids and their complications. Comprehensive historical review Obstet Gynecol Surv 1975 30 01 1 20 1089224 10.1097/00006254-197501000-00001

[16] Tonni G De Felice C Centini G Ginanneschi C Cervical and oral teratoma in the fetus: a systematic review of etiology, pathology, diagnosis, treatment and prognosis Arch Gynecol Obstet 2010 282 04 355 361 20473617 10.1007/s00404-010-1500-7

[17] Neidich M J Prager J D Clark S L Elluru R G Comprehensive airway management of neonatal head and neck teratomas Otolaryngol Head Neck Surg 2011 144 02 257 261 21493427 10.1177/0194599810390012

[18] Catalano P J Urken M L Alvarez M New approach to the management of airway obstruction in “high risk” neonates Arch Otolaryngol Head Neck Surg 1992 118 03 306 309 1554453 10.1001/archotol.1992.01880030094019

[19] Hasiotou M Vakaki M Pitsoulakis G Congenital cervical teratomas Int J Pediatr Otorhinolaryngol 2004 68 09 1133 1139 15302143 10.1016/j.ijporl.2004.04.018

[20] Malhotra S Negi P Sagar P A case of cervical teratoma in an infant Indian J Otolaryngol Head Neck Surg 2022 74 03 6519 6523 36742920 10.1007/s12070-021-02942-w PMC9895199

